# A versatile nano display platform from bacterial spore coat proteins

**DOI:** 10.1038/ncomms7777

**Published:** 2015-04-09

**Authors:** I-Lin Wu, Kedar Narayan, Jean-Philippe Castaing, Fang Tian, Sriram Subramaniam, Kumaran S. Ramamurthi

**Affiliations:** 1 Laboratory of Molecular Biology, National Cancer Institute, National Institutes of Health, Bethesda, Maryland 20892, USA; 2 Laboratory of Cell Biology, National Cancer Institute, National Institutes of Health, Bethesda, Maryland 20892, USA; 3 Department of Biochemistry and Molecular Biology, College of Medicine, Pennsylvania State University, Hershey, Pennsylvania 17033, USA

## Abstract

Dormant bacterial spores are encased in a thick protein shell, the ‘coat’, which contains ∼70 different proteins. The coat protects the spore from environmental insults, and is among the most durable static structures in biology. Owing to extensive cross-linking among coat proteins, this structure has been recalcitrant to detailed biochemical analysis, so molecular details of how it assembles are largely unknown. Here, we reconstitute the basement layer of the coat atop spherical membranes supported by silica beads to create artificial spore-like particles. We report that these synthetic spore husk-encased lipid bilayers (SSHELs) assemble and polymerize into a static structure, mimicking *in vivo* basement layer assembly during sporulation in *Bacillus subtilis*. In addition, we demonstrate that SSHELs may be easily covalently modified with small molecules and proteins. We propose that SSHELs may be versatile display platforms for drugs and vaccines in clinical settings, or for enzymes that neutralize pollutants for environmental remediation.

## Abstract (web summary)

The densely crosslinked protein coats of bacterial spores are among the most durable static structures in biology. Wu *et al.* reconstitute the basement layer of a bacterial spore coat on membrane-coated beads, and generate covalently-modified spore-like particles with therapeutic potential.

## Graphical abstract

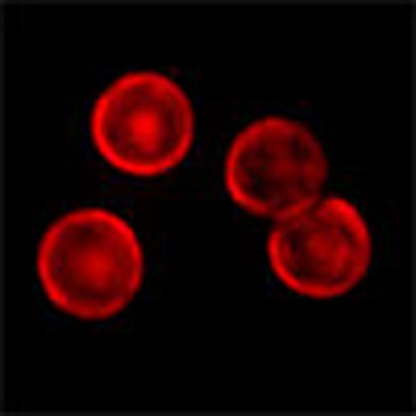

The assembly of static supramolecular structures is a terminal step in morphogenesis. As such, a fundamental challenge in developmental biology is to understand the mechanisms that underlie how large static structures like eggshells and teeth are formed^1^,^2^. A powerful model system to study the construction of such a structure is the bacterial spore coat, an ∼1 μm diameter shell that encases endospores of the Gram-positive bacterium *Bacillus subtilis*. The coat is composed of ∼70 different proteins^3^,^4^ and participates in protecting the spore’s genetic material from environmental insults^5^.

Spore formation initiates when the rod-shaped *B. subtilis* senses the depletion of nutrients in the environment and, instead of dividing by binary fission, divides asymmetrically to produce a smaller daughter cell (the ‘forespore’) and a larger daughter cell (the ‘mother cell’), which are genetically identical, but differentiate to follow separate cell fates^6^,^7^,^8^ (Fig. 1a). Next, the mother cell engulfs the forespore such that the forespore eventually resides in the mother cell cytosol as a double membrane-bound organelle. Ultimately, the mother cell lyses, thereby releasing the mature, now dormant, spore into the environment. During sporulation, coat proteins are synthesized in the mother cell and localize onto the surface of the forespore to form the coat^9^. Coat assembly begins with the construction of a basement layer, which contains a structural protein termed SpoIVA^10^ that displays a multi-domain architecture^11^. The N terminus of SpoIVA binds and hydrolyses ATP^12^,^13^ via a predicted structural fold that resembles the TRAFAC class of P-loop GTPases^14^. ATP hydrolysis drives a structural change in SpoIVA that is required for its irreversible polymerization into a static polymer *in vitro*^13^. SpoIVA is a soluble protein; it is thought to be anchored onto the surface of the developing forespore by SpoVM^15^, a 26-amino-acid amphipathic α-helical protein^16^,^17^ that preferentially embeds onto positively curved membranes with a radius of curvature similar to that of the forespore^18^. *In vivo*, proper assembly of the coat around the forespore absolutely requires SpoIVA and SpoVM^16^,^19^, but it has been unclear if these two proteins are sufficient to initiate coat assembly.

Here, we reconstitute the basement layer of the spore coat atop spherical lipid bilayers supported by silica beads, using purified SpoIVA protein, and synthesized SpoVM peptide to construct synthetic spore husk-encased lipid bilayers (SSHELs). We show that SpoIVA requires SpoVM to uniformly anchor it on the surface of the spherical supported membranes and that stable association of SpoIVA with the beads requires ATP hydrolysis. Interestingly, the behaviour of SpoIVA in the *in vitro* system closely resembled the behaviour of SpoIVA *in vivo* in sporulating *B. subtilis* cells. Examination of the surface of SSHEL particles revealed non-uniformly spaced protrusions that formed a stippled texture only under conditions where SpoIVA polymerized and qualitatively resembled the pitted surface of de-coated spores observed *in vivo*^20^. Finally, we show that SSHELs may be covalently modified, using a click chemistry technology, with small molecules and proteins of interest. We propose that decorated SSHELs may be used as a versatile platform for the display of drugs, enzymes and vaccines.

## Results

### SpoVM is necessary and sufficient to anchor SpoIVA

**Figure 1:**
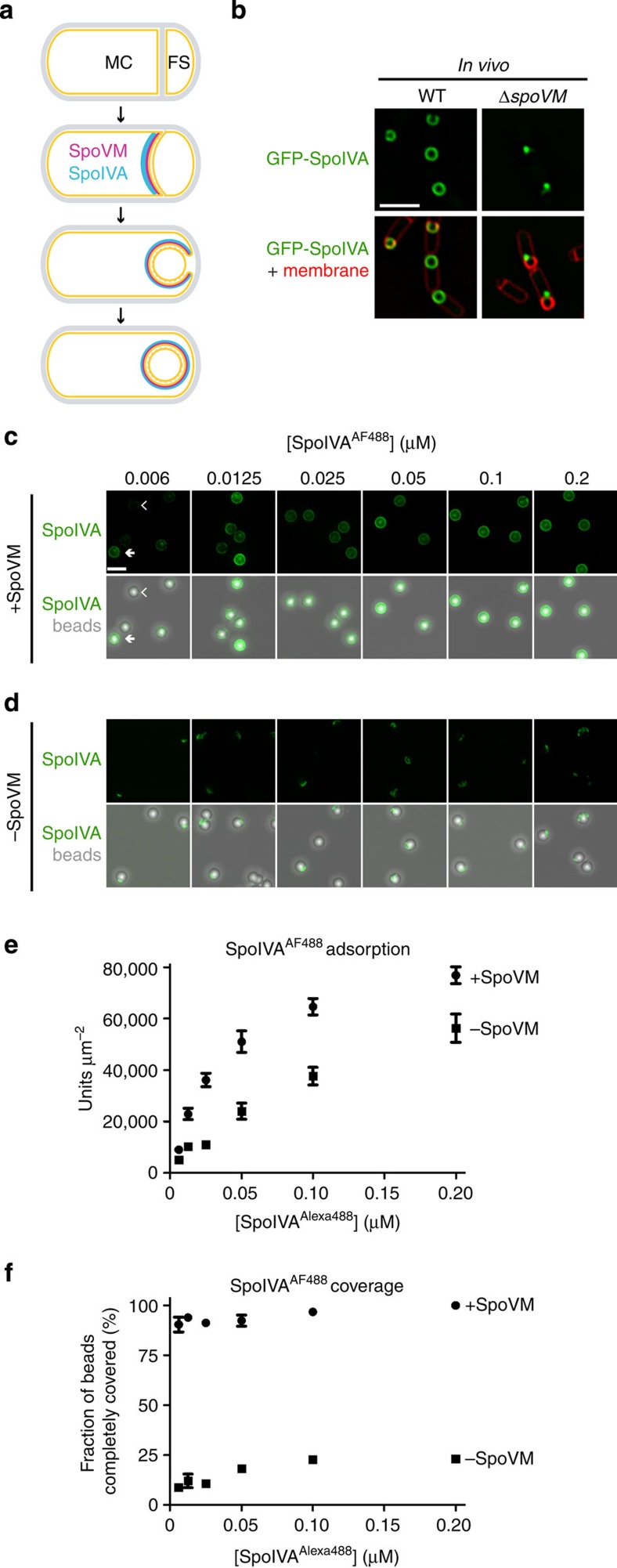
Uniform adsorption of SpoIVA onto SSLBs requires SpoVM. (**a**) Schematic representation of sporulation in *Bacillus subtilis*. Membranes are depicted in yellow; cell wall is depicted in grey. Top: asymmetric division results in the formation of two genetically identical, but differently sized, compartments termed the forespore (FS, which ultimately becomes the mature spore) and the mother cell (MC). Middle panels: the asymmetric septum curves as the mother cell engulfs the forespore. SpoVM molecules (magenta) are produced exclusively in the mother cell and preferentially bind to the positively curved membrane at the engulfing septum. SpoVM recruits SpoIVA (turquoise), also produced exclusively in the mother cell, which polymerizes to form the basement layer of the spore coat. Bottom: eventually, the forespore resides as a double membrane-bound organelle, encased in the basement layer of the spore coat. Additional coat proteins (not depicted) assemble atop the basement layer (**b**) Top: *in vivo* localization of GFP-SpoIVA in sporulating *B. subtilis* cells in the presence (left) or absence (right) of *spoVM*. Bottom: overlay of GFP fluorescence (green) and membranes visualized with the fluorescent dye FM4-64 (red). (**c**,**d**) Concentration-dependent adsorption of AlexaFluor 488-labelled SpoIVA onto SSLBs in the presence (**c**) or absence (**d**) of SpoVM. Overlay of DIC (grey) and AlexaFluor 488 fluoresescence (green) for each panel is shown below. Arrow and arrowhead indicate an SSLB with high and low fluorescence, respectively. Scale bar, 3 μm. (**e**) Mean adsorbance of SpoIVA^AF488^ onto the surface of SSLBs in the presence (●) or absence (■) of SpoVM. Each data point represents at least 35 SSLB particles from three replicate experiments; error bars represent s.e.m. (**f**) Fraction of SSLBs displaying any fluorescence intensity above background level whose pattern of adsorption is qualitatively uniform, in the presence (●) or absence (■) of SpoVM.

Proper localization of SpoIVA *in vivo* depends on SpoVM^21^. In a current model of spore coat basement layer assembly, the hydrophobic SpoVM spontaneously inserts preferentially into convex membranes to mark the forespore surface as the site for coat assembly^22^, whereupon at least one residue in the N terminus of SpoVM directly interacts with a C-terminal SpoIVA residue to recruit and anchor SpoIVA to the surface of the developing forespore^15^. Consistent with this model, in wild-type cells GFP-SpoIVA localized uniformly around the forespore *in vivo* in those cells that had completed engulfment, and as arcs in those cells undergoing engulfment (Fig. 1b)^21^. In contrast, in the absence of SpoVM, GFP-SpoIVA localized instead as a single focus near the mother cell-proximal face of the forespore and failed to encase the forespore^15^,^21^ (Fig. 1b).

To test this model of basement layer assembly *in vitro*, we first constructed spherical supported lipid bilayers (SSLBs) by coating 2-μm-diameter silica beads with a phospholipid biliayer^23^,^24^ to mimic the surface of the forespore. Next, we adsorbed synthesized SpoVM peptide to the SSLBs at concentrations that saturated the surface of the SSLBs. We then purified a cysteine-less variant of SpoIVA that harboured a single engineered cysteine at the N terminus, which we modified with the fluorescent dye AlexaFluor488. *B. subtilis* cells producing this cysteine-less variant of SpoIVA as the only version of SpoIVA sporulated at 109±15% (s.d.; *n*=3) efficiency relative to wild type, indicating that it was largely functional *in vivo*. We then incubated the SpoVM-coated SSLBs with increasing concentrations of SpoIVA^AF488^ and measured its adsorption using fluorescence microscopy (Fig. 1c). At the lowest concentration of SpoIVA^AF488^ that we tested, some beads displayed obvious qualitative fluorescence that was distributed roughly uniformly around the SSLBs (Fig. 1c, arrow), whereas others displayed little or no fluorescence (Fig. 1c, arrowheads). At higher concentrations of SpoIVA^AF488^, the heterogeneity in fluorescence between SSLBs was reduced and SpoIVA^AF488^ adsorption approached saturation (Fig. 1c,e). In the absence of SpoVM, increasing concentrations of SpoIVA^AF488^ again resulted in increasing fluorescence intensity on the SSLBs (Fig. 1e), but the pattern of adsorption was markedly different. Rather than uniform coating of SSLBs, SpoIVA^AF488^ localized as patches on the SSLB surfaces (Fig. 1d) in a manner that was reminiscent of the distribution of GFP-SpoIVA *in vivo* on the surface of the forespore in the absence of SpoVM (Fig. 1b). Quantification of distribution patterns of SpoIVA^AF488^ on multiple SSLBs *in vitro* in the presence and absence of SpoVM revealed that, while approximately 100% of SSLBs were qualitatively encased completely with SpoIVA^AF488^ in the presence of SpoVM at all concentrations of SpoIVA^AF488^ that we tested, less than 20% of SSLBs were encased even at the highest SpoIVA^AF488^ concentration in the absence of SpoVM (Fig. 1f). We conclude that SpoIVA likely has an intrinsic affinity for membranes that allows it to initially localize to the surface of the forespore *in vivo* and to the surface of SSLBs *in vitro*, but that uniform coverage of SpoIVA atop either surface requires the localization of SpoVM to uniformly tether it to the membrane. Further, the similarity in the patterns of SpoIVA adsorption observed *in vivo* and *in vitro* indicates that SpoVM is sufficient for anchoring and uniformly distributing SpoIVA around a spherical membrane surface such as the forespore.

### Stable association of SpoIVA with the forespore requires ATP

**Figure 2:**
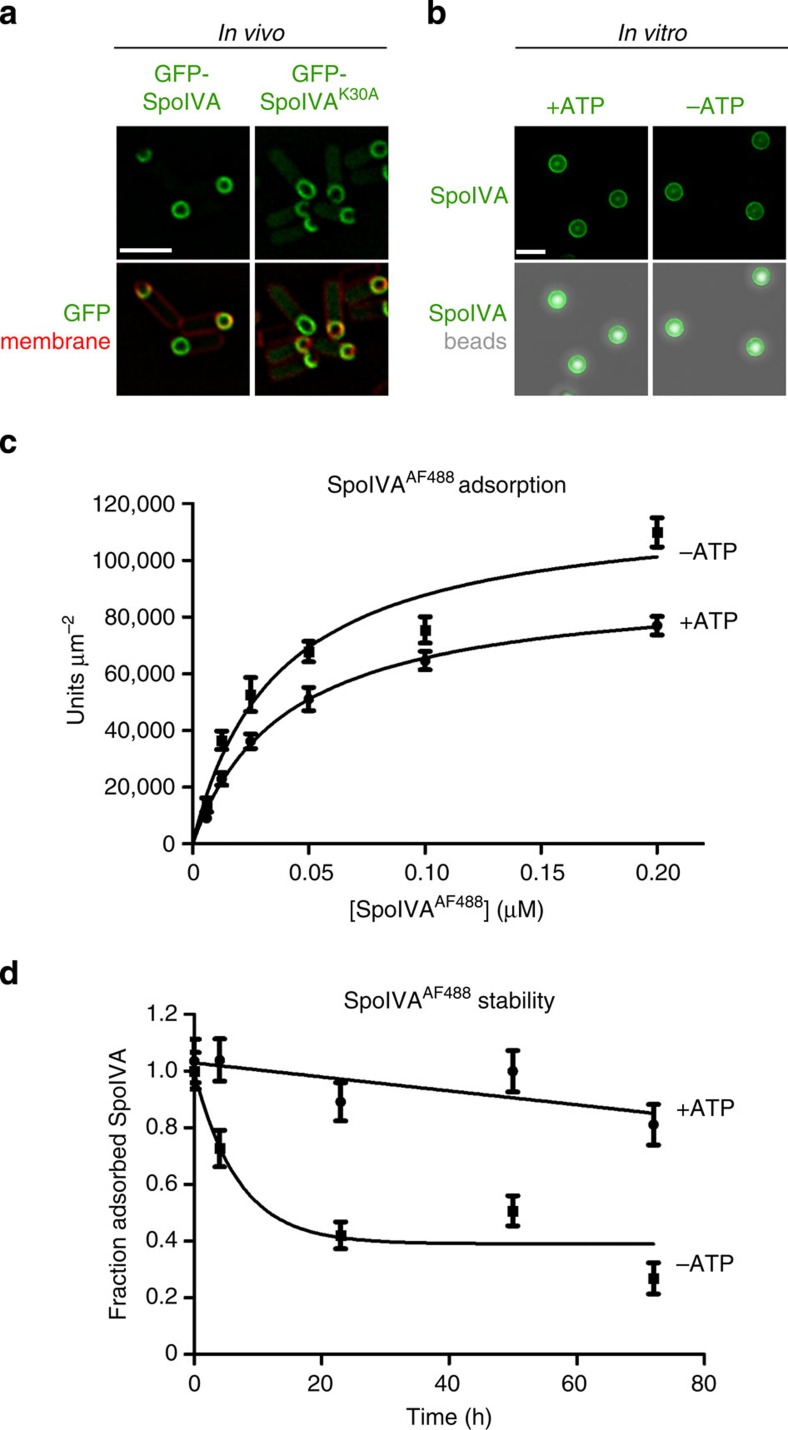
Stable association of SpoIVA on the surface of SSLBs requires ATP. (**a**) *In vivo* localization of GFP-SpoIVA (left) or GFP-SpoIVA^K30A^ (right, which is unable to bind ATP). Bottom: overlay of GFP fluorescence (green) and membranes visualized with FM4-64 (red) as described above. (**b**) Adsorption of SpoIVA^AF488^ *in vitro* onto SSLBs coated with SpoVM in the presence (left) or absence (right) of ATP. Scale bar, 3 μm. (**c**) Concentration-dependent adsorption of SpoIVA^AF488^ onto SSLBs coated with SpoVM in the presence (●) or absence (■) of ATP. (**d**) Retention of SpoIVA^AF488^ on the surface of SSLBs, adsorbed either in the presence (●) or absence (■) of ATP at different time points after competition with exogenously added excess, unlabelled purified SpoIVA. Each data point represents at least 35 SSLB particles from three replicate experiments; error bars represent s.e.m.

Unlike dynamic cytoskeleton proteins^25^,^26^ and static intermediate filaments^27^, the static polymerization of SpoIVA requires both ATP binding and hydrolysis^12^, which drives a conformational change that places the protein in a polymerization-competent state^13^. *In vivo*, GFP-SpoIVA^K30A^, a variant that harbours a disruption in the Walker A motif that abrogates ATP binding, largely localized at the forespore surface, indicated by 71±0.6% (s.e.m.; *n*=50) of the total fluorescence intensity that was associated with the forespore (with 83% (*n*=115) of engulfed forespores completely encased with GFP-SpoIVA^K30A^), compared with 93±0.6% (*n*=50) (with 92% (*n*=122) of engulfed forespores completely encased with GFP-SpoIVA) for GFP-SpoIVA (Fig. 2a)^12^. However, the increased amount of cytosolic GFP-SpoIVA^K30A^ (∼29% of the total for SpoIVA^K30A^ versus only ∼7% for WT SpoIVA) suggested that, in the absence of polymerization, its association with the forespore may be reversible. To investigate the role of ATP in basement layer assembly, we incubated varying concentrations of SpoIVA^AF488^ with SpoVM-coated SSLBs in the presence and absence of ATP and measured its adsorption. At all concentrations tested, SpoIVA^AF488^ adsorption onto SSLBs was similar in the presence and absence of ATP (Fig. 2b,c), similar to the behaviour of GFP-SpoIVA^K30A^ in comparison with GFP-SpoIVA *in vivo* (Fig. 2a). To test whether ATP could be required for the irreversible association of SpoIVA on the membrane, we first adsorbed SpoIVA^AF488^ on the surface of SpoVM-coated SSLBs in the presence or absence of ATP, added an 800-fold excess of unlabelled SpoIVA, then monitored the association of SpoIVA^AF488^ with the SSLBs over time (Fig. 2d). The competition assay revealed that, in the presence of ATP, 81%±7% (*n*>35 SSLBs) of the initial amount of SpoIVA^AF488^ remained adsorbed on the SSLBs even after 72 h, suggesting that polymerized SpoIVA formed a stable shell atop the beads. However, in the absence of ATP, the initially bound SpoIVA^AF488^ was rapidly competed off from SSLBs and only 72%±6% (n>35 SSLBs) remained associated with the SSLBs after just 4 h; after 72 h, only 26%±5% (n>35 SSLBs) remained associated (Fig. 2d), indicating a dynamic exchange between surface-bound SpoIVA^AF488^ and unlabelled SpoIVA in solution. Thus, the kinetic measurement performed *in vitro* demonstrating desorption of SpoIVA^AF488^ in the absence of ATP, likely mimicked the incomplete association of GFP-SpoIVA^K30A^ we observed *in vivo*. We conclude that after SpoVM tethers SpoIVA onto the membrane surface, SpoIVA polymerization, driven by ATP, ensures the static association of the spore coat basement layer on the forespore surface. The data are also consistent with a model^13^ in which recruitment of SpoIVA by SpoVM increases the local concentration of SpoIVA at a membrane surface to exceed the threshold concentration for SpoIVA polymerization, thereby ensuring the preferential polymerization of SpoIVA on the forespore surface, and not elsewhere.

### Ultrastructure of SSHEL particles

**Figure 3:**
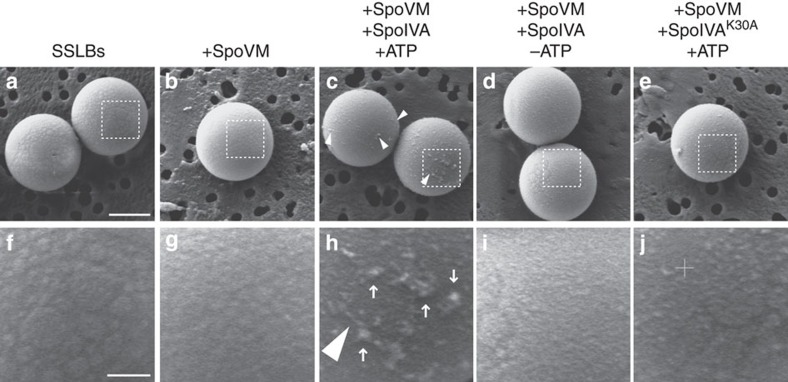
Surface topography of SSHEL particles. Top: scanning electron micrographs of SSLBs (**a**); SSLBs coated with SpoVM (**b**); SSLBs coated with SpoVM and SpoIVA in the presence (**c**) or absence (**d**) of ATP, or coated with SpoVM and SpoIVA^K30A^ in the presence of ATP (**e**). (**f**–**j**) Higher magnification view of indicated areas in **a**–**e**, respectively. Arrows: protrusions; arrowheads: short filaments. Scale bar, 1 μm (**a**–**e**); 250 nm (**f**–**j**).

We previously showed that SpoIVA polymerizes into filaments in solution in the presence of ATP^12^,^13^, but its detailed ultrastructure upon assembly on a two-dimensional surface has not been reported. In addition, although several recent studies have employed atomic force microscopy to visualize the different layers of the coat in cells of mutants arrested at particular stages of coat assembly^20^,^28^,^29^, it has been difficult to identify which proteins make up which particular feature in the context of the milieu of proteins in the coat, a problem that is amplified when examining the basement layer that is buried under the other layers of the coat. Since the behaviour of SpoIVA recruitment and stability in our *in vitro* system mimicked that of SpoIVA *in vivo*, we examined the topography of SSHEL particles by scanning electron microscopy (SEM) to understand the ultrastructure of the basement layer of the coat. In the absence of any proteins, the surfaces of SSLBs were largely smooth, displaying only characteristic shallow ridges formed by membranes when viewed by SEM (Fig. 3a,f). Addition of SpoVM alone did not significantly alter the surface of the SSLBs (Fig. 3b,g). However, on addition of SpoVM and SpoIVA in the presence of ATP, the surface of the beads assumed a more rough appearance (Fig. 3c). Closer examination of these surfaces (Fig. 3h) revealed non-uniformly shaped protrusions that were spaced irregularly (Fig. 3h, arrows), which were qualitatively reminiscent of the ‘pitted’ surface reported on the surface of mature mutant spores (Δ*spoIVD*) examined by AFM that did not assemble outer layers of the coat^20^. Interestingly, these surfaces also frequently displayed short filaments (Fig. 3c,h; arrowheads) that were reminiscent of SpoIVA filaments detected by transmission electron microscopy that formed in solution in the presence of ATP^12^. In contrast, the surface of SSLBs incubated with SpoVM and SpoIVA in the absence of ATP (Fig. 3d,i), or SSLBs incubated with SpoVM, SpoIVA^K30A^ and ATP (Fig. 3e,j) did not display such features. Taken together, we conclude that the SSHEL particles we have constructed harbour a static polymerized protein shell that displays a qualitatively differently textured surface than one that simply contains adsorbed proteins.

### Covalent decoration of SSHELs with molecules of interest

**Figure 4:**
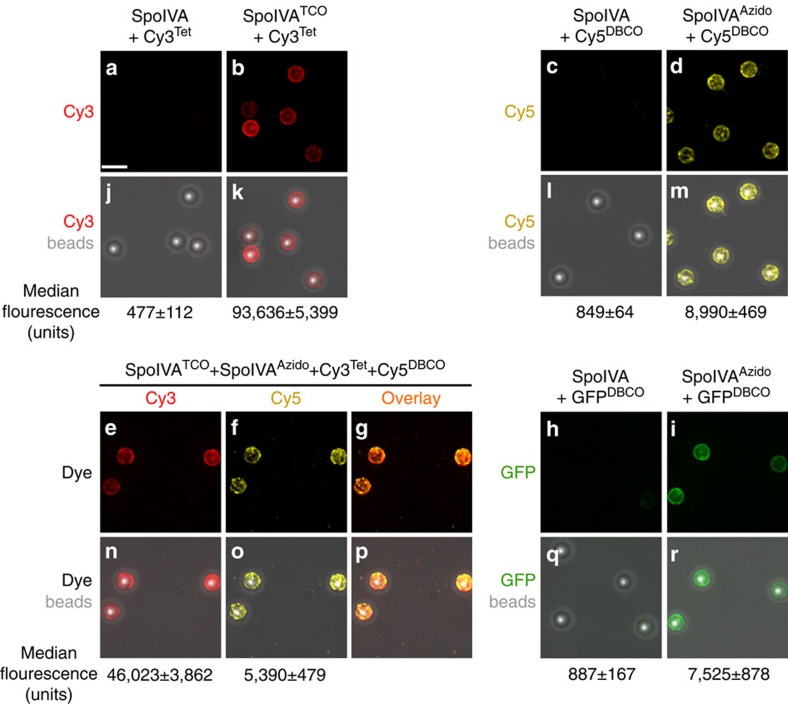
Covalent modification of the surface of SSHEL particles with small molecules or protein of interest. (**a**,**b**) Modification of the surface of SSHEL particles, with tetrazine-labelled Cy3 fluorophore, decorated with either (**a**) SpoIVA or (**b**) SpoIVA labelled with TCO-PEG_3_. (**c**,**d**) Modification of the surface of SSHEL particles, with DBCO-labelled Cy5 fluorophore, decorated with either (**c**) SpoIVA or (**d**) SpoIVA labelled with Azido-PEG_3_. (**e**–**g**) Stepwise modification of SSHEL particles, with Cy3^Tet^ and Cy5^DBCO^, decorated with SpoIVA^TCO^ and SpoIVA^Azido^, viewed using the Cy3 filter (**e**) or the Cy5 filter (**f**). (**g**) Overlay of (**e**) and (**f**). (**h**,**i**) Modification of SSHEL particles, with DBCO-labelled GFP, decorated with SpoIVA (**h**) or SpoIVA^Azido^ (**i**). (**j**–**r**) Overlay of DIC (grey) and fluorescence from (**a**–**i**), respectively. Median fluorescence intensity of decorated SSHEL particles (arbitrary units) is displayed below each panel. Scale bar, 3 μm. Median fluorescence intensities were determined from three replicate experiments; error is s.e.m. (*n*>40).

Bacterial spore surfaces have been reported to be modified with a variety of proteins, and the use of spores modified in this manner has been proposed as a display system for ligands of interest that may be used as vaccine display platforms and for drug delivery^30^,^31^,^32^,^33^, for the display of enzymes to neutralize environmental pollution^34^,^35^ and to screen for novel binding partners^36^. However, these techniques often rely on the use of genetically modified organisms and, as they are built on a viable spore, contain thousands of extraneous factors that, depending on the situation, could potentially interfere with the function of a displayed molecule of interest. As SpoVM and SpoIVA alone were able to assemble into a stable shell *in vitro* with a distinct morphology whose behaviour mimicked the coat basement layer *in vivo*, we sought to covalently link small molecules and proteins to the surface of SSHEL particles using copper-free click chemistry^37^,^38^. To this end, we first assembled SSHEL particles using a cysteine-less variant of SpoIVA harbouring a single engineered cysteine at the N terminus that was modified with *trans*-cyclooctene (TCO), which could be selectively labelled by tetrazine^39^. Incubation of tetrazine-labelled fluorescent dye Cy3 (Cy3^Tet^) with SSHEL particles constructed with SpoIVA without modification by TCO did not result in appreciable fluorescence, but SSHEL particles decorated with TCO-modified SpoIVA were able to be decorated with Cy3^Tet^ (Fig. 4a,b,j,k). Similarly, SSHEL particles constructed with SpoIVA modified with azide was specifically able to be conjugated with the fluorescent dye Cy5 modified with the cognate click molecule dibenzocyclooctynes (DBCO)^40^ (Fig. 4c,d,l,m). To test whether multiple molecules may be clicked onto the surface of SSHELs, we first constructed SSHEL particles with a mix of SpoIVA modified with either TCO or azide, and then incubated them stepwise with Cy3^Tet^ and Cy5^DBCO^. When viewed under different microscope filters to detect Cy3 and Cy5 fluorescence, the same SSHEL particles were labelled with both Cy3^Tet^ and Cy5^DBCO^ (Fig. 4e-g,n-p), each at roughly half the fluorescence intensity as SSHELs constructed with a single modified version of SpoIVA (Fig. 4b,d), indicating that SSHEL particles may display at least two different covalently attached molecules using this strategy. To test whether a protein of interest may be similarly covalently linked to the surface of SSHELs, we first conjugated green fluorescent protein (GFP) with DBCO. Whereas SSHEL particles constructed with unmodified SpoIVA displayed minimal fluorescence, the fluorescence from SSHEL particles constructed with SpoIVA^Azido^ was almost ninefold higher, indicating that proteins of interest may be specifically and covalently coupled to the surface. We therefore propose that SSHEL particles, composed of a minimal defined set of components, may be covalently decorated with a combination of small molecules and proteins of interest and may serve as an alternate display platform for spore-based vaccines, biocatalysts or drug delivery.

## Discussion

Spore formation in *Bacillus subtilis* is an attractive model system to elucidate mechanisms that underlie morphogenesis. However, to reconstitute morphogenetic events *in vitro* is difficult due to the complexity of a living organism. We demonstrated here that we are now able to recapitulate the initiation of spore coat assembly atop SSLBs with defined protein components to build what we term SSHEL particles. We envision that this system may be used as a robust *in vitro* assay to study the morphogenesis of complex structures such as the spore coat and to test the specific proposed predictions concerning the network of protein–protein interactions in the spore coat^41^ beyond the basement layer.

The use of spore display using *in vivo*-modified bacterial spores has been proposed as a unique molecular adjuvant with emergent interests for mucosal vaccine design^42^,^43^ and for the display of enzymes that aid in environmental remediation efforts^34^,^35^. We suggest that SSHEL particles covalently decorated with multiple specific small molecules and proteins of interest may be used as an additional strategy for these efforts as they provide several potential benefits. First, SSHEL particles are of defined composition, so their surfaces are devoid of extraneous (potentially uncharacterized) proteins that may interfere with a specific function of a displayed protein or molecule. Second, reconstruction of the display platform *in vitro* eschews the use of a living, potentially genetically modified, organism capable of replication or horizontal gene transfer in the final product. Finally, by employing an *in vitro* system, we envision that the density of a single displayed molecule may be finely tuned by adjusting the ratio of modifiable and unmodifiable SpoIVA used to construct SSHEL particles. This may be particularly useful, for example, when the magnitude of an immune response may be sensitive to the density of a particular antigen. In addition, current technologies permit the display of multiple ligands on the surface of *B. subtilis* spores, for example, by incorporating a streptavidin-fused coat protein on the spore surface, which can then interact with multiple biotin-conjugated molecules of interest^44^. Using the system described here, we envision that a large number of molecules may be specifically and covalently displayed on the surface of SSHEL particles, and that this number is limited only by the number of orthogonal conjugation molecules available to perform the click chemistry reactions. Moreover, the relative ratios of the displayed molecules on a bead may be precisely adjusted simply by adjusting the ratios of specifically modified SpoIVA molecules used to construct the SSHELs. Finally, while the external display of molecules presents several potential technological opportunities, we envision that, ultimately, the assembly of synthetic spore coats modified with ligands of interest atop large lipid vesicles may allow for the osmotically stable encasement of high amounts of cargo molecules of interest that could be delivered to tissue-specific locations.

## Methods

### Strain construction

All *B. subtilis* strains are isogeneic derivatives of PY79 (ref. 45). Strains KR160 (*thrC::gfp-spoIVA spec*), KR178 (Δ*spoVM::tetR thrC::gfp-spoIVA spec*) and KR394 (*thrC::gfp-spoIVA*^*K30A*^ *spec*), and construction of His-tagged SpoIVA^K30A^ have been described^12^,^15^. A His_6_-tagged cysteine-less SpoIVA variant (C98S; we substituted with Ser because it occurs at this position in other SpoIVA orthologues^13^) with an extra cysteine engineered into the N terminus was constructed by the QuikChange Lightning Site-Directed Mutagenesis kit (Agilent) using plasmid pKR145 (ref. 12) as the template to produce plasmid pJP120. Plasmid pIL3, encoding superfolder GFP (sfGFP) for purification, was PCR amplified from pBAD24-sfGFPX1 (ref. 46) and cloned into pET28a (Novagen) using *Nhe*I and *Hind*III restriction sites. Surface-exposed cysteine (S147C)^47^ was introduced into sfGFP by site-directed mutagenesis using pIL3 as a template to generate pIL4.

### Protein purification and labelling

SpoIVA and sfGFP variants were overproduced in *E. coli* BL21(DE3) and purified using Ni^2+^ affinity chromatography (Qiagen)^13^. SpoIVA was additionally purified by ion-exchange chromatography (MonoQ; Pharmacia)^13^. SpoIVA was labelled with Alexa Fluor 488 C5-maleimide (Life Technologies) following the manufacturer’s protocol. For click chemistry conjugation, SpoIVA and sfGFP were labelled with *Trans*-Cyclooctene-PEG_3_-Maleimide, Azido-PEG_3_-Maleimide Kit or DBCO-PEG_4_-Maleimide as described by the manufacturer (Click Chemistry Tools). In brief, 20-fold molar excess of maleimide reagent was added to the protein samples and incubated for overnight at 4 °C, and the excess reagent was removed by PD-10 desalting column (GE Healthcare).

### SSLB preparation

SSLBs were made largely as described^23^,^24^. In brief, liposomes were produced by the sonication method using 100 μl (10 mg ml^−1^) *E. coli* polar lipid extract (Avanti) that were first evaporated under vacuum overnight at room temperature and hydrated in 1 ml ultrapure water. Resuspended lipids were subjected to five freeze-thaw cycles between methanol-dry ice bath and 42 °C water bath, followed by sonication until the suspension became transparent. Debris was removed by centrifugation at 13,000*g* for 10 min, and the supernatant containing unilamellar vesicles was retained. Silica beads (2 μm, 10 mg ml^−1^) (PolySciences) were prepared for coating by washing three times each in 1 ml ultrapure water, followed by methanol and 1 M NaOH. The beads were rinsed and resuspended in 200 μl ultrapure water. The SSLBs were constructed by mixing the silica beads with 200 μl prepared liposomes and 1 mM CaCl_2_, and incubated at 42 °C for 30 min. After vortexing, SSLBs were collected by centrifugation at 13,000*g* for 1 min, washed three times with ultrapure water and resuspended in 1 ml buffer A (50 mM Tris and 400 mM NaCl at pH7.5).

### SSHEL particle construction

SpoVM was synthesized as 26-amino-acid peptide (Biomatik Corp.) and incubated at 10 μM (final concentration) with 2.5 mg ml^−1^ 2-μm-diameter SSLBs in buffer A, overnight at 25 °C following a programme of alternate shaking and resting every 5 min. SpoVM-coated SSLBs were collected by centrifugation at 13,000*g* for 1 min, and then were incubated with varying concentrations of SpoIVA^AF488^ in a final volume of 100 μl buffer A containing 10 mM MgCl_2_ and 4 mM ATP, overnight at room temperature with gentle inversion. SSHEL particles were collected by centrifugation and resuspended in 100 μl buffer A for microscopy. For competition assays, SpoVM-SSLBs were incubated with 0.006 μM SpoIVA^AF488^ in the presence or absence of 4 mM ATP in buffer A containing 10 mM MgCl_2_. The fluorescent SSLBs were collected by centrifugation and resuspended with 100 μl buffer A containing 5 μM unlabelled SpoIVA. Five-microlitre aliquots were taken at indicated time points for microscopy.

### Epifluorescence microscopy

Overnight cultures of *B. subtilis* harbouring GFP-SpoIVA and variants were induced to sporulate by the resuspension method^48^ in medium containing 1 μg ml^−1^ of the fluorescent membrane dye FM4-64 (Life Technologies). Cells were harvested and prepared for microscopy using a 1% agarose pad made with distilled water and viewed with a DeltaVision Core microscope system (Applied Precision)^49^. Images were captured with a Photometrics Coolsnap HQ2 camera and deconvolved using SoftWorx software (Applied Precision). ImageJ was used to quantify the fluorescence located in the cells and forespores. For microscopy of SSLBs, 5-μl suspensions were placed on a glass bottom culture dish (Mattek Corp.) and covered by the agarose pad as described above. Thirty planes were acquired every 0.2 μm at room temperature; the data were deconvolved using SoftWorx software. The fluorescence intensities were then projected onto a single plane, quantified using SoftWorx software and reported as fluorescence micron^−2^ of SSLB surface area.

### Scanning electron microscopy

SSHEL particles were washed with PBS and fixed in 4% formaldehyde, 2% glutaraldehyde in 0.1 M cacodylate buffer and post fixed using a 1% osmium tetroxide solution. They were then dehydrated in a series of graded alcohols and air dried after a final dehydration course of tetramethylsilane. The samples were subsequently coated with a thin layer of Au/Pd using an EMITECH K575X high resolution sputter coater set at 5 mA deposition current and imaged in a Zeiss NVision40 at a working distance of 5.0–8.2 mm; the SEM was operated at 3 keV landing energy and secondary electrons were recorded at the SE2 detector. The images were acquired with a fast dwell time of 50 ns with × 10 line averaging and at a pixel sampling of 5 nm.

### Surface modification of SSHEL particles

Clickable SSHEL particles were constructed by incubating SpoVM-SSLBs with 0.2 μM SpoIVA^TCO^ or SpoIVA^Azido^ as described above. SSHEL particles were collected by centrifugation and resuspended in click buffer (50 mM Tris, 150 mM NaCl at pH7.5) containing 1 μM Cy3-Tetrazine or Cy5-DBCO (Click Chemistry Tools), inverting at room temperature for 2 h. Dye-coupled SSHEL particles were collected by centrifugation, washed and resuspended in click buffer, and a 5-μl aliquot was taken for microscopy. For stepwise ligation, SSHEL particles were constructed with 0.1 μM SpoIVA^TCO^ and 0.1 μM SpoIVA^Azido^ as described above, collected and resuspended in click buffer with 0.5 μM Cy3-Tetrazine and incubated as described above. Cy3-SSHEL particles were collected and resuspended in click buffer containing 0.5 μM Cy5-DBCO and incubated similarly. The dual-fluorescent SSHELs were collected, washed and resuspended in click buffer for microscopy. To incorporate sfGFP onto SSHEL particles, 0.3 μM sfGFP^DBCO^ was added into SpoIVA^Azido^-SSHEL particles in click buffer containing 1 mM TCEP (Sigma) to prevent non-specific binding and incubated at room temperature for 1 h. The sfGFP coupled-SSHEL particles were collected and resuspended with click buffer inverting at room temperature for 1 h to wash off non-specific binding. The particles were then collected by centrifugation and resuspended in click buffer for microscopy.

## Additional information

**How to cite this article:** Wu, I.-L. *et al.* A versatile nano display platform from bacterial spore coat proteins. *Nat. Commun.* 6:6777 doi: 10.1038/ncomms7777 (2015).
